# Formagen

**Published:** 1898-02

**Authors:** Abraham Konitz


ARTICLE IV.

### FORMAGEN.

BY DR. ABRAHAM KONITZ.

Reported by George Randorf, Berlin, Germany.

Formaldehyde is derived from methylic alcohol, out of which it is obtained as a colorless, odorous gas through incomplete oxidation. Through the researches of myself, Dr. Lepkowski., Krau and others, it is known that formaldehyde has such an effect on an inflamed pulp that the affected tooth can be filled at one sitting. It is used as an aqueous solution of forty per cent. A piece of cotton saturated with this solution, is applied to the inflamed pulp. By this treatment the desired effect is obtained, but the method is faulty in that the formaldehyde solution causes in the pulp, as well as mucous membrane, very severe pain for several hours. Dr. Lepkowski recommends this method only in cases where lack of time demands it.

Hence the question arose, how to obtain the desired result without pain. Many experiments followed, and Lepkowski assures us that weakening and mixing with cocaine is ineffective. After a series of experiments, I found that the required result could be obtained with pure liquefied carbolic acid, which has the property of uniting clear with the formalin in every form.

After long experiments, which I ended in the spring 1885, and which brought no satisfactory result, I tried and made use of formaldehyde in the treatment of roots. I prepared a very spongy cement, such as is commonly used for the capping of inflamed pulps. After many trials, I found a cement especially fit for the purpose, which contains marble and eugenol. In both fluid and powder, the freshly produced formaldehyde is introduced while the two are kept separate. By mixing this fluid and powder on a glass slab, the putty like paste called Formagen is obtained. This is applied to the exposed pulp or, if it is still covered, over the softened dentine. The most violent pulpitis ceases in a few minutes, and the parts harden in about five minutes, so that the tooth can be filled with cement or amalgam.

In the course of somewhat over nine months, there have occurred more than a thousand cases of such teeth which have been successfully treated with Formagen, so that they were filled permanently at one sitting. It is asked if the teeth so treated have been preserved with pulps dead or alive. This question is answered by the fact that the pulp after four or six weeks treatment with Formagen fully recovers and thenceforward performs its normal functions. In teeth which have been treated with Formagen, the fillings as well as the layers of Formagen have been removed, and in every case the exposed pulp has responded with normal sensitiveness.

It is daily asked in what manner the Formagen acts upon the teeth. To this question there cannot yet be any definite answer; but the theory so far as it has been determined is as follows: the pulps covered with Formagen are influenced first by the eugenol, and also by the carbolic acid contained in the fluid, which thus deadens the pain. And during the action of the eugenol, the formaldehyde passes gradually out of the Formagen and penetrates into the pulp, and changes the same in a small region around the exposed place. The developing gases and fluid from the pulp chamber are absorbed by the porous putty. And from the effect of the formaldehyde the pulp becomes hard and tough. The parts of the pulp not affected, meanwhile act normally; and from this fact, and from the action of the formaldehyde upon the pulp, it becomes, for the first days after application of Formagen, inactive, but in a few weeks is restored to its normal functions.—*Items of Interest.*

